# Preliminary Analysis of Collar Sensors for Guide Dog Training Using Convolutional Long Short-Term Memory, Kernel Principal Component Analysis and Multi-Sensor Data Fusion

**DOI:** 10.3390/ani14233403

**Published:** 2024-11-26

**Authors:** Devon Martin, David L. Roberts, Alper Bozkurt

**Affiliations:** 1 Department of Electrical Engineering, North Carolina State University, 890 Oval Dr., Raleigh, NC 27695, USA; 2 Department of Computer Science, North Carolina State University, Campus Box 8206, 890 Oval Drive, Raleigh, NC 27695, USA; robertsd@csc.ncsu.edu

**Keywords:** Conv-LSTM, KPCA, autoencoder, manifold learning, pattern recognition, guide dogs

## Abstract

Guide dogs play a crucial role in enhancing independence and mobility for people with visual impairment, offering invaluable assistance in navigating daily tasks and environments. However, the extensive training required for these dogs is costly, resulting in a limited availability that does not meet the high demand for such skilled working animals. Towards optimizing the training process and to better understand the challenges these guide dogs may be experiencing in the field, we have created a multi-sensor smart collar system. In this study, we developed and compared two supervised machine learning methods to analyze the data acquired from these sensors. We found that the Convolutional Long Short-Term Memory (Conv-LSTM) network worked much more efficiently on subsampled data and Kernel Principal Component Analysis (KPCA) on interpolated data. Each attained approximately 40% accuracy on a 10-state system. Not needing training, KPCA is a much faster method, but not as efficient with larger datasets. Among various sensors on the collar system, we observed that the inertial measurement units account for the vast majority of predictability, and that the addition of environmental acoustic sensing data slightly improved performance in most datasets. We also created a lexicon of data patterns using an unsupervised autoencoder. We present several regions of relatively higher density in the latent variable space that correspond to more common patterns and our attempt to visualize these patterns. In this preliminary effort, we found that several test states could be combined into larger superstates to simplify the testing procedures. Additionally, environmental sensor data did not carry much weight, as air conditioning units maintained the testing room at standard conditions.

## Simple Summary

Guide dogs play a critical role in assisting individuals with visual impairments, enhancing their daily activities and overall quality of life. However, the high cost of guide dog training limits accessibility for many within this community. Incorporating custom-designed sensors and advanced data analytics into early-stage training has the potential to improve training success rates and reduce associated costs. In this study, we developed a custom sensor system that attaches to guide dog puppy collars to collect motion, audio, and environmental data during the “In-For-Training” evaluation process at a leading guide dog school in the United States. We employed two primary machine learning methods capable of making predictions based on various combinations of smart collar sensor data.

## 1. Introduction

There were over 7.5 million non-institutionalized visually disabled people in the United States alone in 2016 [1]. Guide dogs are used to help these people in everyday life, but not every dog is suitable for this critical role. Less than 1% of these visually impaired people in the United States have access to a guide dog. The major barrier is the cost of training a guide dog for a visually impaired person, which is close to fifty to seventy thousand US dollars [2,3]. This is exacerbated by the high dropout rate of guide dogs during training. More than half of dogs in training will fail out of the program to become working guide dogs [4]. Even following a guide dog school graduation, there is a small yet noticeable dropout rate throughout performance years until retirement after approximately 8.5 years [5]. However, some attempts to reduce dropout rate have worked previously. For example, about 30% of guide dogs used to be disqualified because of skeletal hip issues, but this was reduced to negligible thanks to breeding programs in the late 1990s [6]. As another example, potential guide dogs can be returned for retraining if they originally failed [7]. Of the 40% of the returned dogs, 53% later graduated, improving the overall graduation rate.

A specific potential guide dog’s personality and temperament are the greatest obstacles towards graduation. Personality is defined as behavioral tendencies that are consistent within an individual, but which are distinguishable from others. At The Seeing Eye, one of the largest guide dog schools in the US, personality-related behavioral problems constituted approximately 21% of dogs failing training the first time. Of personality-related problems, the most prominent issue was fearfulness [7]. Fear experienced during traumatizing early puppyhood events leads to general behavioral problems within dogs [8] and is a significant predictor of guide dog training failure [9]. At Guiding Eyes for the Blind, these traits are tested as a “Fear of Strangers” score evaluated by professionals [10]. Another common challenge is the susceptibility to distractions [7]. A guide dog needs heightened attention to street signs and other daily activities without being overly attentive to loud noises like a car horn. The noise and other-dog distraction tests are temperament tests designed to evaluate distractedness early [11] and similar tests are performed in pre-evaluations for guide dog training.

While breeds, genetics, and certain biological aspects can be well controlled through breeding programs and systematic medical assessment, it is also important to evaluate the early developmental progress. Our team developed and used wearable devices in several applications of working dog behavior monitoring [10,12,13,14,15], including sleep monitoring, temperament evaluations, and breathing and heart rate assessment.

For potential guide dog puppies, we formerly presented a smart collar system [16,17,18] capable of recording both dog behavioral and immediate environmental data continuously. The collar records body movement using an inertial measurement unit (IMU) and barking behavior using sound sensors. Movement of the body is expected to be the greatest behavioral predictor since excessive motions can indicate excitability or a proneness to being easily distracted, whereas minimal movement can indicate passivity or fear responses. This can help identify dogs that are unsuitable to work as guide dogs. The sound sensors can similarly assist by identifying behaviors such as barking for attention-seeking or identifying sudden noises that may overly startle the dog. Immediate surrounding environmental information, such as ambient temperature and light, relative humidity, and barometric pressure, are also registered. These sensors have been assessed to identify exposure to new places during early puppy raising [16]. In the context of this present work, these are not expected to vary significantly, though may still improve our predictions.

While this collar system can be used in various stages of the guide dog training, in this study, we focused on analyzing the data collected from these devices during a behavioral evaluation protocol performed in a guide dog school. In particular, we deployed this system on puppies undergoing the In-For-Training (IFT) Evaluation protocol in one of the largest guide dog schools in the US (Guiding Eyes for the Blind (GEB), Yorktown Heights, NY, USA) for determining dog fitness before entering their official training program.

The rest of this paper is organized as follows: we introduce the broader goals of this study and then detail our data analysis methods by describing the dataset. We also provide details about our two supervised methods, Convolutional Long Short-Term Memory (Conv-LSTM) network and Kernel Principal Component Analysis (PCA), and our unsupervised method, the autoencoder (AE). Then, we describe the performance and results of each of these methods. Lastly, we conclude by comparing and discussing the accuracy of these two supervised methods, and benchmark with what the autoencoder was able to extract from our dataset.

## 2. Motivation of the Study

We describe the motivation of this study in three sections. First, we discuss how this research is intended to improve on prior research using smart collars on guide dogs. Then, we share how this study would identify certain behaviors and apply to future guide dog testing while also reviewing the state-of-the-practice in dog behavior tests. Lastly, we focus on the particular objective of this study to develop advanced data analytics to process smart collar data acquired during IFT testing.

### 2.1. The Unique Contribution of This Study for Smart Collar-Based Guide Dog Performance Assessment

This work follows and expands on prior work with smart collars on guide dogs to transform subjective temperament scoring into an objective data-based assessment in several ways by performing the following:

- Using new data collected during IFTs, which had not been available before.
- Accessing a significantly larger dataset than earlier works as a significant step towards scaling up.
- Analyzing the data acquired in-lab under controlled conditions as a baseline, allowing better expectations of full-scale field data when acquired in the future.

We formerly presented a smart collar system capable of continuously recording both dog behavioral and immediate environmental data. The development of these smart collar devices emphasizes two specific goals. The first is to acquire objective measurements of guide dog behavior while the puppies are trained and evaluated in the guide dog school [10,14]. Traditionally, more common subjective scoring is performed by a trained staff member using the behavioral checklist (BCL) or other questionnaires. Even with trained staff, these scoring methods are subjective and more liable to higher error rates. Therefore, there is an opportunity to use objective, quantitative data and data processing methods to enhance behavior testing. Secondly, as part of their training process, guide dog schools transfer potential guide dog puppies to volunteer raisers who care for the puppies at their homes until a certain age. This period is a major blind spot during early dog development and these collars with sensors are able to help alleviate uncertainty by providing continuous monitoring of behaviors in the variety of environments that the puppies enter under the care of volunteer raisers [16,18]. Holder et al. [17] outline how participatory research combined with wearable electronic sensor systems can help to investigate this significant period in the life of a guide dog puppy [17]. This current work is a new step to this overall exploration to attain and use objective quantitative measures to assess guide dog performance. Within this big picture, we particularly focus on IFT measurements in this presented study.

In guide dog schools, identifying the puppies that are not perfectly suited to work as a guide dog is a key strategy to minimize training costs. Guide dog training programs focus on balancing the uncertainty of early testing to make decisions as soon as possible with the higher opportunity costs of later and more accurate testing. It is suggested that testing should take place around the 14-month mark because behavioral traits are more stable at this age [11]. Our prior work focused on early puppy temperament scoring, where we acquired data from more than five hundred puppies using a smart harness system resulting in the largest ever dataset from this population [10,14,16,18]. The current work presented in this paper focuses on the testing performed on puppies at the 15–18-month age, which may have made it more likely for us to extract features representative of future adult behavior.

### 2.2. Identifying Dog Temperament

This section provides a more detailed view of how we seek to examine dog temperament as a part of our main overarching goal. A large-scale survey (1851 total) on dog behavioral problems reported by dog owners revealed eleven main factors [8], including aggression, fear, separation anxiety, and excitability. For guide dogs specifically, ref. [11] lists common tests that can be used to identify desirable temperaments. The most relevant are the six temperament tests, including a passive test, where time to relax is recorded; a noise test, where a food bowl is dropped to distract the dog; and a dog distraction test, where an unfamiliar dog is presented. In line with these tests, GEB performs such tests during its IFT process for prospective guide dogs. These tests are performed sequentially and additionally include distracting events, such as an umbrella opening, activating a vacuum cleaner, and shaking a metal nail-filled can. The main objective of the IFT is to monitor a dog’s tendency to be distracted and observe levels of fear and excitability. These are recorded in a checklist with scores from 1 to 5.

The smart collars developed by our team have previously been shown to distinguish various activities in a controlled training and temperament evaluation environment [16]. In this current study, the number of states (each corresponding to IFT tests) was expanded to 50, where the smart collar data were analyzed to observe subtle differences between certain temperamental behaviors. The states in the IFT could correspond directly to certain dog temperaments. For example, a highly excitable dog would be expected to have more erratic movement and show high jerkiness in collected inertial measurement data. Similarly, the passive test [11] can be inferred from the “Explore-Enters Space” state, when the dog is allowed to roam the test environment, as well as the “Ignore dog” state, when the trainer ignores the dog. Movement during this “Ignore dog” state could indicate excitability or separation anxiety. This is a preliminary analysis to set up a baseline and investigate whether we can extract features and patterns on smart collar data that correspond to temperament demonstrated during various IFT states.

### 2.3. Exploring Various Data Analysis Methods for Smart Collar IFT Data Analysis

**Figure 1:**
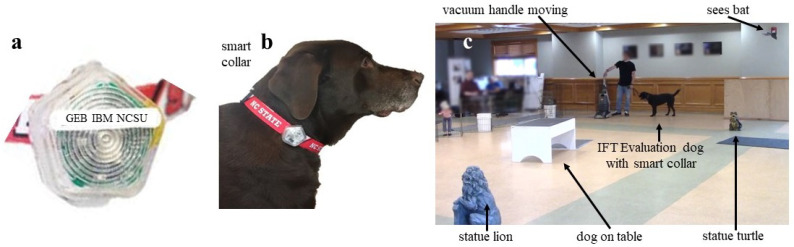
(**a**) Smart collar device, (**b**) smart collar implemented, and (**c**) dog undergoing IFT with smart collar on. Examples of objects corresponding to labels are shown in (**c**).

To reiterate, the IFT evaluation is one of the most important evaluation steps conducted on 15–18-month-old dogs after an approximately 12-month socialization process with volunteer raisers and when they arrive to the guide dog school, to determine whether they are a fit for guide training or not. For such an evaluation, temperament and rates of adaptability to strangers, other dogs, and new environments are examined (see Figure 1). Trainers seek well-posed, confident dogs for further guide dog training [19] as guide dogs. For the last two years, our smart collar system has been deployed during the IFT tests conducted at the GEB guide dog school to support this study [17]. The collar would be connected to an accompanying custom iOS app over Bluetooth. The graphical user interface of the app allowed trainers to record the timing of controlled stimuli used as test events. This created a time-accurate label that could be associated with the collar’s data collection. The exploration of dog behavior captured in the data was aimed to determine subtle reactions to the evaluation steps while also assessing the environmental parameters. This is expected to add objectivity to the test evaluations, which qualify or disqualify a given dog more accurately and earlier in the training process.

Our smart collar system uses multiple different sensors to collect data related to the dog’s behavior and environment; we focused on employing the most accurate data fusion within the scope of this study to attain higher accuracy than using individual sensors. Data fusion architectures are numerous and highly specific to the problem being analyzed and format of the data [20,21,22,23,24,25]. Therefore, one major project goal was to find and optimize such an architecture specific to monitoring canine behavior within the context of the immediate environment. Additionally, because data were collected as a time series for each sensor, we anticipated large temporal dependencies. While there are already many time series models such as the common Auto-Regressive Integrated Moving Average (ARIMA), these lack any data fusion capabilities. We instead chose Long Short-term Memory Networks (LSTMs), which can accommodate fusion from multiple sensors and have shown strong predictability on time series data [26].

Another goal of this study was to simplify future dog behavioral evaluations using this newly acquired data. We observed that the behavior categories classified under the IFT evaluation were towards the reactions demonstrated to the applied stimuli while other natural and generic dog behaviors were not labeled. For instance, a dog may jump at any random time during a pre-defined evaluation step. While this behavior may not be paid attention to or categorized directly under the IFT, it is a quantifiable and easily recognizable event that could be identified using machine learning and used to further analyze the dog’s behavior. For this reason, we were interested in and included an unsupervised learning approach to categorize these ill-defined, dog-specific tendencies extracted from the data directly. For this, we performed manifold learning for the categorization of common patterns of the sensor data [27].

Overall, the specific goals we would like to achieve with this study include the following:

- A more accurate understanding of canine behavior in response to the IFT test;
- Accounting for individual effects like temperament on behavior;
- Apply multi-sensor data fusion to understand dog temperament and behavior;
- Explore creating a lexicon for in-field behavior interpretation.

## 3. Methodology

This section describes the dataset used and preprocessing procedures, followed by the design of the supervised machine learning approaches: LSTMs and KPCA. Finally, an unsupervised method, autoencoders, is introduced for creating a data lexicon of guide dog actions and behaviors.

### 3.1. Dataset Description

IFT sessions are conducted roughly every 1–2 months at Guiding Eyes for the Blind guide dog school facilities. Typically, between 15 and 30 dogs undergo the IFT testing during each session, each lasting about ten minutes. Data from these sessions have been accrued for the last two years, creating a dataset of 460 unique dogs tested. During the session, the dog being evaluated to work as a guide dog was presented with various stimuli such as an examination by a veterinarian and suddenly turning on a vacuum cleaner. These are some events that may commonly create anxiety or other emotional responses in dogs in general. The precise start and stop times of these events were recorded by an associated trainer using our iOS app developed specifically for IFT evaluations [17].

During this evaluation, the collar-based sensor system collected behavioral data using inertial measurement units (IMUs) to assess body movement, and a microphone to detect loud barking sounds. While we describe it as an audio sensor, it actually only collects decibel levels at a much lower sampling rate than speech for privacy concerns. These sound levels can come from the dog or the environment. Meanwhile, environment-only conditions were recorded using ambient light, ambient temperature, relative humidity, and barometric pressure sensors. Because different physical signals change with a different rate, all the sensors had different sampling rates. In general, the IMUs, audio, and light sensors were sampled at a much higher rate (100 Hz) than the slowly changing temperature, humidity, and pressure sensors (1 Hz). This adjustment is required to conserve battery power and the bandwidth of the Bluetooth Low Energy protocol used for wireless transmission of the data.

Interpolation was required to account for the different sampling rates of the different sensors. The IFT test sequentially proceeded through a total of 50 different labeled events, including passing by objects or ignoring loud noises. We think of these as fifty labeled ’states’ moving forward. However, many other studies that seek to classify motion used much fewer states, typically five to ten. For a better comparison with other motion classifier studies, we selected a smaller 10-state dataset as well. Based on preliminary models similar to those later described, these ten states were chosen from the most easily distinguishable fifty states (the states with the greatest confusion matrix scores). Lastly, for data fusion, we considered using the IMU alone (similar to other studies), IMU with audio, and IMU with audio and with environmental sensors (temperature, humidity, etc.). Note that because of the low sampling rate of the environmental sensors, there were not enough data to meaningfully create the two 50-state IMU-Audio-Environmental datasets.

Table 1 shows the total of 10 datasets that were assembled. The datasets are differentiated by whether the data were interpolated, the size of the state-space, and what sensors were included:

- Interpolation or subset;
- A 50-state or 10-state classification;
- IMU or IMU-Audio or IMU-Audio-Env.

### 3.2. Data Preprocessing

Interpolation was required to account for the differences in sampling rates. We performed a linear interpolation to bring lower sampling rate sensors in line with higher ones, creating 500-length sequences for all sensors. These are the “interpolate” sampling datasets. We then took these and split them into twenty smaller sequences each of length 25. These are the “subsample” sampling datasets. The subsample datasets therefore have twenty times the data samples, but of much shorter duration. Data were then grouped by the times of the IFT activities, providing a many-to-one relationship between the data and labels. We then took the interpolated data and performed standard Z-normalization to reduce the number of later execution times and computation complexity. This was performed for each sensor modality, m, throughout an entire IFT session.

$$Z_{m,i}=\frac{X_{m,i}-\mu_{m}}{\sigma_{m}}$$

### 3.3. Supervised Methods

Using supervised learning, our aim was to be able to classify canine behavior from on-board collar sensors using LSTMs, where the ground truth is the observation of the human trainer as labeled in the app. Previous experiments have shown that deep convolutional LSTMs have been successful for multimodal activity recognition using accelerometry [28,29]. Accelerometry and ECG have also been previously used for guide dog evaluations using convolutional networks and LSTMs [10]. However, these former tests considered fewer and more similar sensors. In this study, we fused accelerometry, audio, and environmental sensor data.

#### 3.3.1. Description of LSTM Models

Prior research has shown that LSTMs attain great performance when working with sequential data because they were specifically designed to track long-term data patterns. Therefore, we fit and evaluated LSTM models for label predictions for our dataset. Subsequent hyperparameter investigations on LSTMs [26] revealed that the learning rate is the most important hyperparameter for LSTM models, followed by hidden layer size, with most other hyperparameters being generally negligent. We therefore focused primarily on the learning rate and hidden layer sizes when tuning our Conv-LSTM model.

Depending on the specific dataset, the model may have slight variants. For the 10-state models, we finished the model with a 10-node dense layer, while for the 50-state model, we finished the model with a 50-node dense layer. The number of channels also varied depending on which sensors were used (IMU, audio, and environmental sensors with 3, 1, and 4 channels, respectively). We also considered using a higher-capacity model using convolution layers of 1024 instead of 64 for the interpolated datasets.

**Figure 2:**
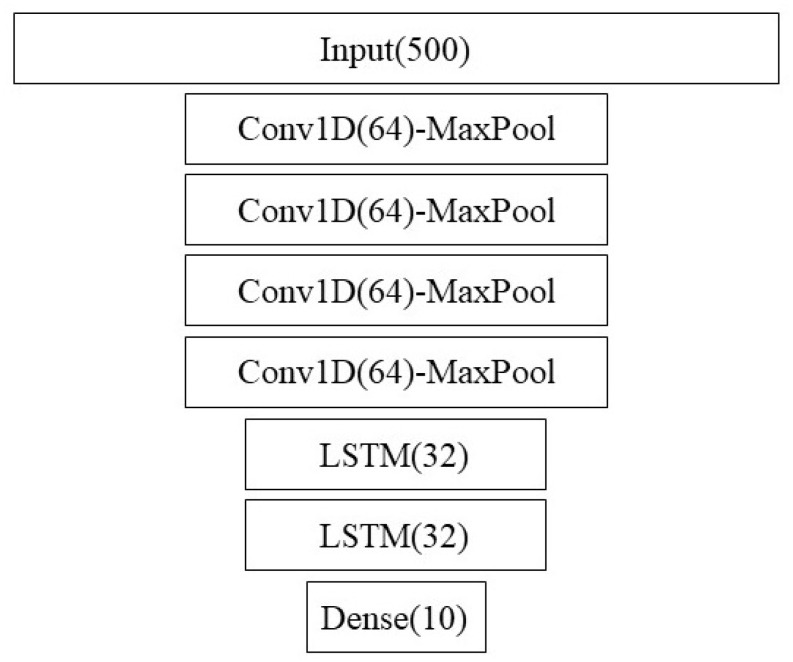
ConvLSTM architecture strongly based on [29].

Figure 2 shows the architecture of the convolutional LSTM model. Note that we also used this structure using 1024 nodes instead of 64 as a larger-capacity model. The results were similar though.

The LSTM models were trained in North Carolina State University’s high-performance computing cluster node Hazel with an attached GPU. Depending on availability, we either used a single Intel Xeon 1.8 GHz core with NVIDIA GeForce GTX 1080 or a single Intel Xeon 2.6 GHz core with NVIDIA GeForce RTX 2080 Ti.

#### 3.3.2. Description of KPCA Models and Setup

**Figure 3:**
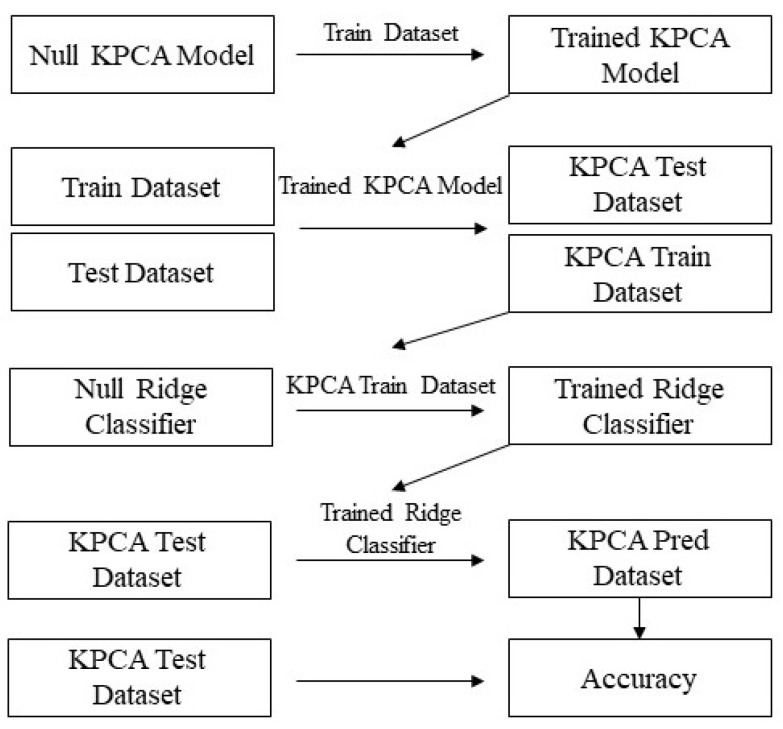
KPCA architecture.

Figure 3 shows the setup of our KPCA method. PCA works on input data (*X*) only and is typically used as a dimensional reduction technique. We split data into a 70/30 train/test split. Then, we used the training data to train the KPCA model, after which we obtained the KPCA components for both the train and test data. Following this, we used the training KPCA components and corresponding training classifications to train a Ridge classifier. The Ridge classifier then penalized less useful components and this was how dimensional reduction was performed. The test classifications were then acquired using the trained Ridge classifier and test KPCA components. KPCA had one main unidimensional hyperparameter: γ. In addition to this optimization, only linear training for the Ridge classifier was required, giving this method a huge complexity advantage over the Conv-LSTM model.

### 3.4. Unsupervised Methods

Our use of Conv-LSTMs and KPCA targeted the behavior prediction during various IFT tasks/steps used to evaluate suitability for guide dog training. We explored whether, at least to some degree, behavioral actions may be determined by monitoring IMUs, audio and immediate environmental conditions. It is also desirable to generalize the data patterns for a more accurate behavior-to-data dictionary for exploring the patterns beyond the IFT. Usually, pattern learning experiments are conducted in well-controlled environments and with well-practiced actions, such as running on a treadmill with a pre-set protocol. This configuration largely limits many real-world effects such as personality differences, mistakes in performance and corresponding corrections, and large amounts of uncontrolled noise in the signal. Even with this in mind, however, we hypothesize that certain actions will still be prominent, such as a dog jumping randomly or sitting for a period of time, and that these should be distinguishable. Note also that these behaviors are not necessarily correlated with labeled data from the IFT sessions, warranting unsupervised learning approaches.

We attempted several multivariate unsupervised learning methods to label certain consistent and repetitive behaviors observable within the data from the collar-based sensor system. The challenge was that we could not segment the data space into subspaces, as we expected high amounts of spatial overlap between actions. Instead, we were interested in identifying patterns within sequential time segments of the data. For this purpose, we specifically looked at manifold learning and autoencoders.

#### 3.4.1. Manifold Learning

Manifold learning is a general method for learning a dictionary of patterns in the data. This approach seeks to find a low-dimensional space from data that exist in a high-dimensional space [27,30]. A given datapoint in our datasets has at most eight dimensions, $x\in R^{8}$, accounting for IMUs, audio, and environmental signal streams. However, incorporating sequences for pattern recognition results in a much higher dimensionality. With n sequential points, $x\in R^{8^{n}}$. This makes manifold learning more useful in this context.

**Figure 4:**
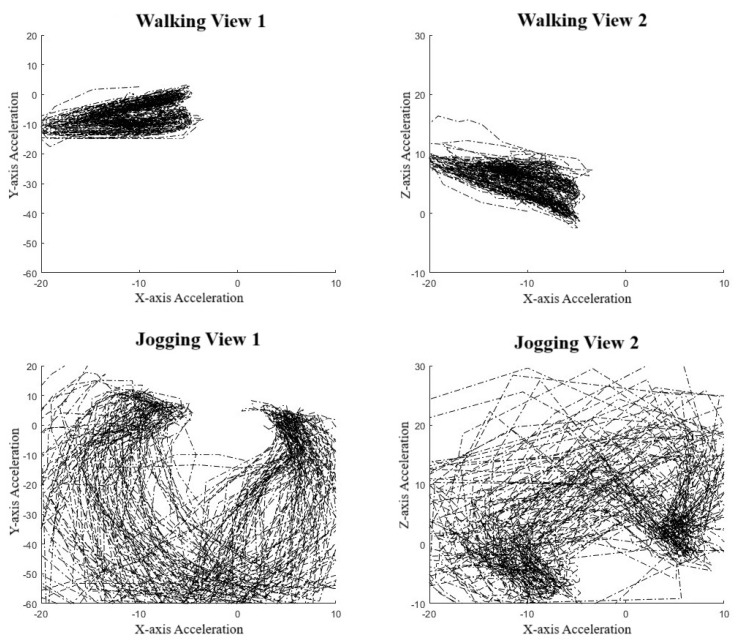
Manifold against action. Top figures are walking cycles and bottom figures are jogging cycles. Left figures show *Y*-axis against *X*-axis and the right is an alternative view showing *Z*-axis against *X*-axis [31].

**Figure 5:**
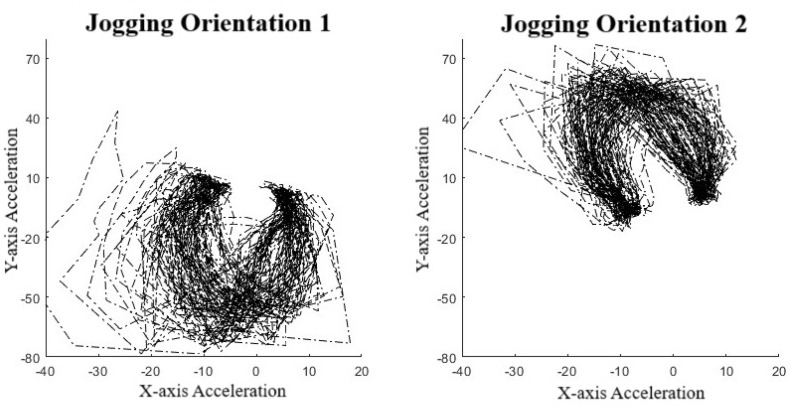
Effects of orientation on manifolds.

To validate the use of manifold learning in this context, we performed a simple preliminary test using our collar-based system to ensure that we could observe real-world manifolds. With one collar in hand, we performed some exercises, including simply walking and jogging around a short path. We observed that for the IMU data (Figure 4), we could easily distinguish two manifolds, one corresponding to walking, the other to jogging. These plots display lines in order to highlight the data sequence patterns rather than the datapoints themselves. Noticeably, the walking manifold takes up a much smaller overall space than the jogging manifold. The jogging manifold has a U shape with higher variation in the center but consistency at the ends. Jogging and walking are easily distinguished based on the shape. We also repeated the experiment with a different collar orientation since IMU sensor values are orientation-specific. Figure 5 shows this effect on jogging, where we see that the data pattern looks to have been flipped over the *x*-axis. However, the pattern within the dataset has remained the same as an inverted U shape. This suggests that the manifolds should be more abstract to account for rotations and translations.

Clearly, different movement patterns during gait are easily distinguishable by their corresponding manifolds when using data acquired from our collar sensor system, at least in this controlled setting. As a simple proof-of-concept demonstration, this analysis indicates that various motions are distinguishable. In the context of the IFT, as an example, a dog with a confident, smooth trot is likely to be distinguished from a nervous dog pulling on a leash. However, the data quality and controls from the IFT sessions are far less and therefore expected to be noisier, potentially producing large-variance manifolds. This is an expected difficulty when creating a dictionary of manifolds.

After producing a working manifold dictionary set, we can then use it to classify new patterns. Given a pattern, we can determine the distance from each manifold in the set and assign it to the closest one. We can project a new data pattern onto the manifolds and the one with the lowest reconstruction error is classified as the behavior associated with this manifold.

#### 3.4.2. Autoencoder

Autoencoders (AEs) are great and popular unsupervised learning models that simultaneously achieve excellent dimensional reduction outcomes. AEs are a type of neural net architectures with two segments: (1) an encoder where hidden layers become subsequently smaller until a minuscule feature space, or latent representation, of only a few traits is reached, and (2) a decoder, which performs the opposite operation from the latent space back up to the original size of the dataset. Loss is calculated from the difference between the original and reconstructed data. Using AEs, only the most important characteristics of the data are learned in the latent space.

**Figure 6:**
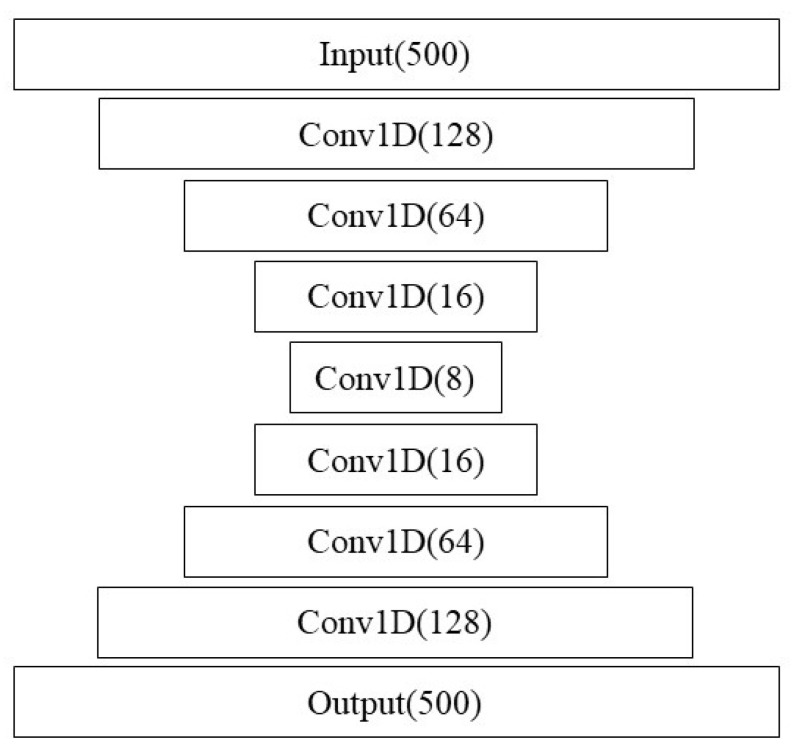
Autoencoder architecture.

Vanilla AEs are not designed to handle sequential data, though decent results for sequences can be obtained using convolutions. For this reason, we considered Conv-AEs, where encoding and decoding layers are constructed out of layers of 1D Conv units (Figure 6). Data are windowed with a heavy overlap, and chains of convolution layers are used for encoding and decoding. Additionally, potentially, since this AE setup uses a similar structure to the Conv-LSTM networks outlined earlier for IFT classification, there is the potential for transfer learning being used between the supervised and unsupervised algorithms.

As before, depending on availability, we either used a single Intel Xeon 1.8 GHz core with NVIDIA GeForce GTX 1080 or a single Intel Xeon 2.6 GHz core with NVIDIA GeForce RTX 2080 Ti.

#### 3.4.3. Unsupervised Interpretation and the Pre-Image Problem

We hypothesize that the unsupervised learning methods should identify a much smaller, important subspace of the patterns in our dog dataset. This subspace should be much smaller than a given pattern’s space while discriminating between different manifold classes. In the case of KPCA, this space is called the Reproducing Kernel Hilbert space (RKHS) [32] and with the LSTM-AE, it is the latent variable space. In using unsupervised methods, we were not sure which input patterns would prove to be the most common. Knowing these common patterns would add much interpretability to our findings. The autoencoder framework converges the input space into a latent space, but for the interpretability of these latent space clusters, we are interested in the inverse problem, finding the common patterns. This is known more formally as the pre-image problem [30,32].

To formalize this problem, we consider an input, *x*, from an input space, $\chi \in R^{M}$, and latent variable, ϕ, from space $\Phi \in R^{N}$ with M>>N. Let ϕ(x) be the mapping provided by an LSTM-AE, KPCA, or other function estimation method. With these terms defined, our goal is to first identify an optimal $\phi^{*}\in \Phi$ that represents a high-density latent-space region. We then want to find the corresponding *x* whose mapping ϕ(x) can reach as close as possible in some optimal sense to our $\phi^{*}$. A reasonable objective function to represent this is to solve the following:

(1) $$x^{*}=\arg\min_{x\in \chi }||\phi \left(x\right)-\phi^{*}{\left|\right|}^{2}.$$

For the first problem of selecting a $\phi^{*}$, we can use clustering methods within the latent space. This is accomplished with methods like K-means or elliptic classification. The clustering can be selected by unsupervised approaches using the Kernel density estimation in Φ. Alternatively, supervised versions can be used, either by grouping ϕ(x) that share a category or by grouping ϕ(x) that share a category and are correctly predicted in the validation set. From these clusters, an initial choice of $\phi^{*}$ is the centroid of the cluster. This would provide a representative of the behavior in the input space. As a second approach, a series of cluster edge points could be selected and used to find the corresponding variances of the behavior in the pre-image space χ.

For the second problem of finding $x^{*}$, we can obtain an initial estimate using a $x_{0}$ with $\phi (x_{0})$ near $\phi^{*}$. From here, gradient descent or other optimization approaches can be used constrained by being in the space around the *x*s that have ϕ(x)s around $\phi^{*}$. However, since we have developed an autoencoder, the decoder section can be used as the mapping from Φ to *X*, naturally solving the pre-image problem. Nevertheless, we use this terminology moving forward.

## 4. Results

This section presents the results of the Conv-LSTM network, performance of the KPCA method, and the performance of the autoencoder technique.

### 4.1. Conv-LSTM Results

**Figure 7:**
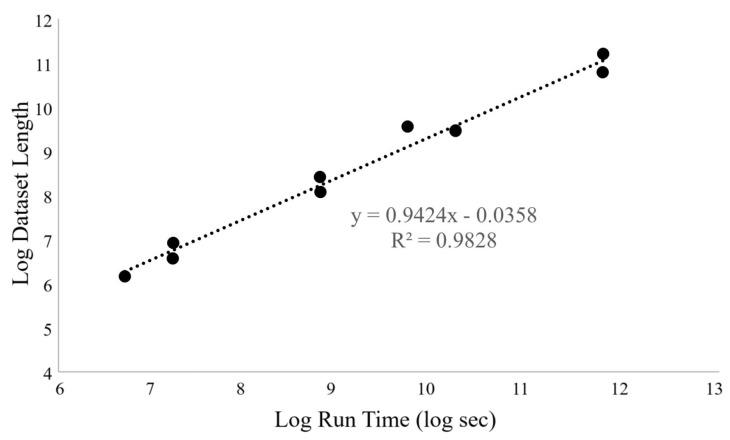
Relation between LSTM train time and dataset length.

The average memory usage for all the datasets was constant at 1.89 ± 0.3 GB. This was expected to be constant since neural networks load data in chunks. Run time depended linearly on dataset length (number of entries), as shown in Figure 7. This suggests a strong linear relationship between dataset size and performance, as expected.

**Figure 8:**
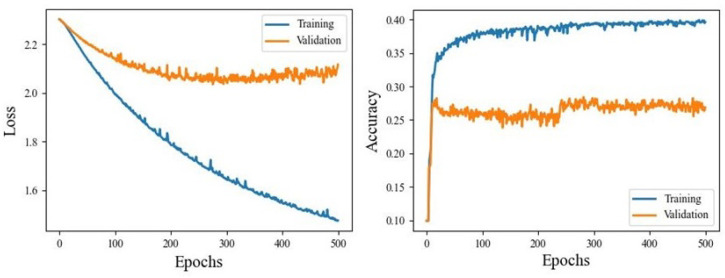
LSTM training curves for interpolated 10-state IMU-only dataset.

Figure 8 shows an example loss and accuracy training and validation curves for one of the datasets. Losses show that validation loss achieved a minimum of around 250 epochs while there was some overfitting of the training data throughout all epochs. Validation accuracy, however, showed a fast convergence at about 50 epochs, but had a stepwise improvement around 250 epochs. This is undesirable in general because stepwise improvements are not smooth and therefore unpredictable. However, the magnitude of the step was only a few percent, and so could be reasonably ignored. Again, the training curve showed a decent amount of overfitting.

Table 2 compares the overall performances of the Conv-LSTM network on each of the ten datasets. We show macro precision, recall, F1-score, and accuracy for each dataset. Note that the 50-state IMU-Audio-Env datasets were not tested because there were insufficient data for each state.

**Figure 9:**
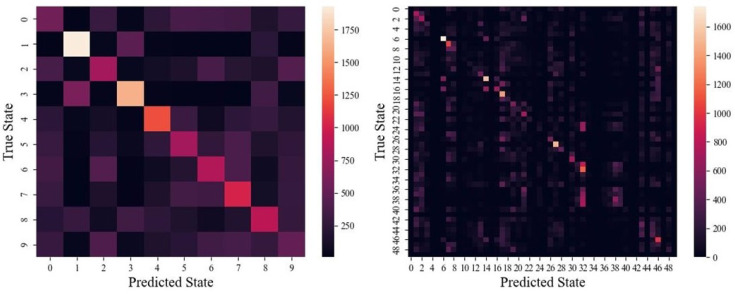
Examples of (**left**) the 10-state confusion matrix and (**right**) a 50-state confusion matrix for Conv-LSTM.

Lastly, example confusion matrices are shown in Figure 9.

### 4.2. KPCA Results

**Figure 10:**
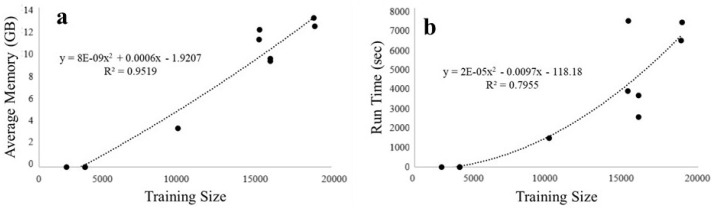
Relation between KPCA training (**a**) space vs. dataset length and (**b**) time vs. dataset length.

For KPCA, we had to subsample some of the datasets in order to comply with high-performance computing space requirements. Most datasets were small enough to not require special requirements, but the subsampled datasets had too much data to run. These were therefore subsampled to either a quarter or a sixteenth of their original sizes. The average memory usage and run times for KPCA on all the datasets showed an accelerating increase in requirements against training size, as shown in Figure 10. The time complexity of calculating the Kernel matrix in KPCA is $O(n^{3})$, and so, our findings are in line with theory. Some of the smaller tested datasets were able to be calculated in several minutes while larger ones took days and 51 GB of memory.

Table 3 compares the overall performances of the KPCA method on each of the ten datasets. We show macro precision, recall, F1-score, and accuracy for each dataset. Note again that the 50-state IMU-Audio-Environmental datasets were not tested because there were insufficient data for each canine state.

### 4.3. IFT State Space Stability

**Figure 11:**
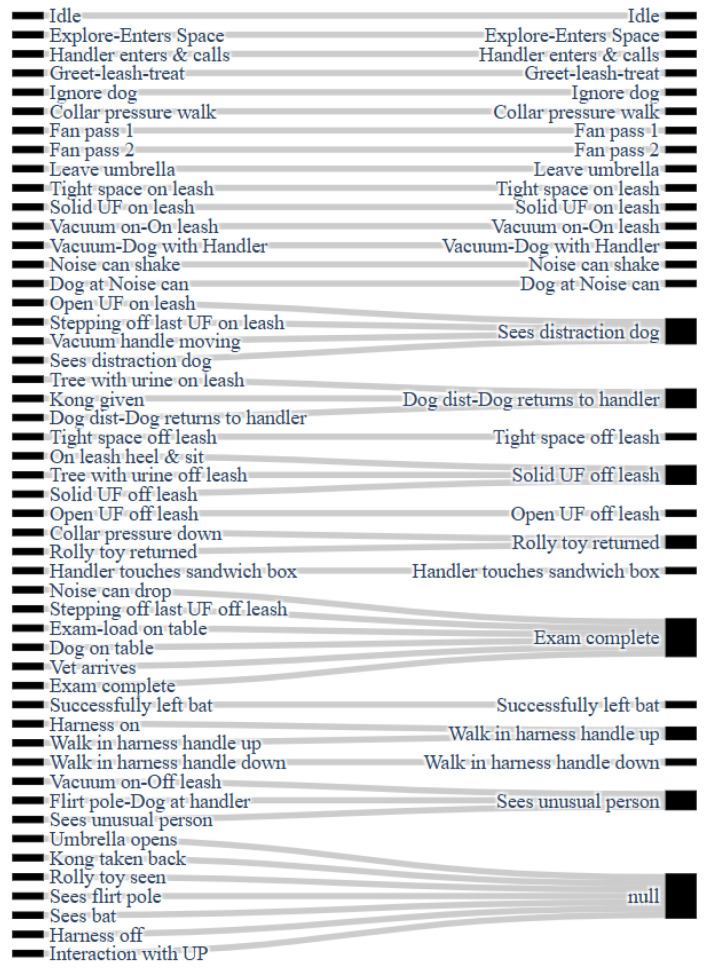
Sankey diagram showing common IFT state confusion. The null state indicates confusion with many other states.

We were interested in understanding the confusion between IFT states. We note that each model (ConvLSTM and KPCA) produces a most-common state, but that this can mistakenly correspond to another state. To visualize this, Figure 11 shows a Sankey diagram. On the left are all 50 states; on the right are the states that are most commonly selected from the states on the left. Occasionally, an IFT state is confused evenly across many other states; in this case, these are associated with the null state.

### 4.4. Autoencoder in Latent Variable Space

**Figure 12:**
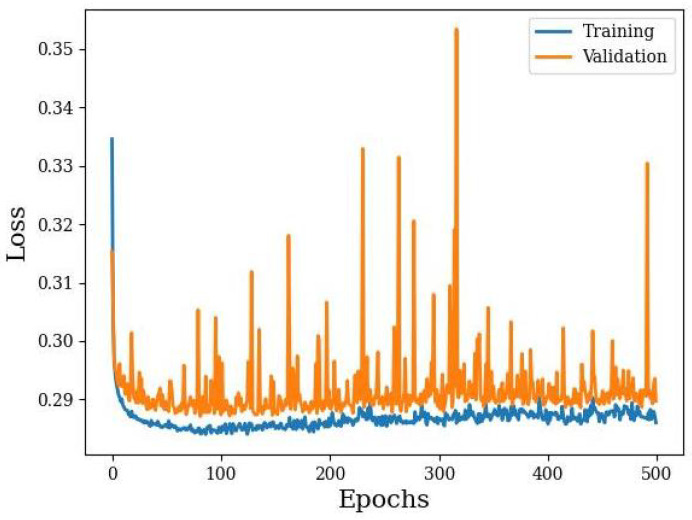
Autoencoder training of the interpolated 10-state IMU-only dataset.

The autoencoder was used to establish a much smaller latent variable space that corresponded to common data patterns obtained from the dogs. We see the training curves for the interpolated 10-state IMU-only dataset in Figure 12 as an example. The training converged to a validation loss minimum of about 0.29 after about 70 epochs. Unfortunately, the validation loss shows a number of irregular spikes in loss, suggesting a level of instability. This instability does not exist in the training set.

**Figure 13:**
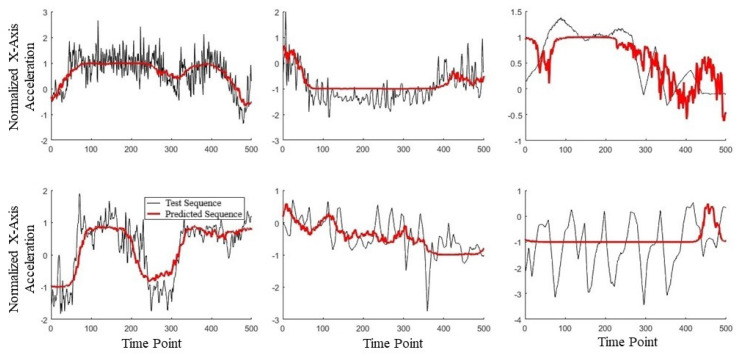
Six examples of autoencoder input sequence (black) and corresponding output sequence (red) from the interpolate-50-IMU dataset.

Following training, we compared several input and output sequences to check whether the model was learning patterns. Example sequences can be seen in Figure 13, with black showing the original sequence and red the recreated sequence. In the leftmost four plots, the autoencoder had successfully learned low-frequency, low-order patterns that look like smoothed versions of the original. The two plots on the right, however, show a stark mismatch between the original and the recreated. The top-right shows a higher-frequency section in the recreated signal and the bottom-right shows a lack of any decent fit for the oscillatory sequence. Overall, this would indicate that most of the sequences are being reasonably estimated (at least the overall trend), while a significant minority of cases are undetermined.

## 5. Discussion

The default accuracy, if the system were random, for the 50-state system is just 2% and for the 10-state system, 10%. So, the 15% accuracy on the 50-state system and 30–40% accuracy on the 10-state system is considered to be very promising. While some other recent work in the literature evaluating predictions from IMU data have higher reported accuracies (80% to 100%), it is important to indicate that these solve a noticeably simpler problem [28,33,34]. For instance, each of these have a much smaller number of states (typically 4 to 6), as opposed to our 10 or 50 states. Our data are also much noisier for a number of practical reasons.

1. The timing of the different states is not concrete; these can vary depending on the trainer using the app or how the dog is performing that day.
2. The states we used were not designed to be different and were created for a standardized protocol; as a result, many states are similar to each other, such as ones during which distractions are provided for the dog.
3. The sensors are attached to the collar and is not firmly connected to the dog; this produces motion artifacts that especially affect IMU performance.
4. Dogs are less obedient at following protocol than human subjects.

A couple of confusion matrices are shown in Figure 9. In both cases, two trends indicating a good fit emerge. First, a noticeable diagonal means that predicted states agree with the true state. And the second, that most predicted states have some non-zero background, suggests that the model is indeed learning most of the states to some extent. However, the 50-state confusion matrix does show a lack of some predicted states, likely due to sparsity in the importance vs. discernibility of these states. Recalling that each state makes up either 2% of the 50-state or 10% of the 10-state datasets, there is no concern of overfitting to an overrepresented class in this case.

Looking at the accuracy data in Table 2 and Table 3, several instances are observed in which combining the data from multiple sensors showed a substantial improvement in performance. The Conv-LSTM network on the subsampled 10-state datasets showed a marked increase in performance from 38%, using only the IMU, to 39% when incorporating audio data, then to 47% with the environmental data. The Conv-LSTM network on the subsampled 50-state datasets also showed a minor improvement from 15.0% using the IMU-only to 15.3% when including audio data. Interestingly, data fusion showed worse or ambiguous results for the Conv-LSTM network on the interpolated datasets. Accuracy for the interpolated 10-state system was consistently around 30% while on the interpolated 50-state datasets, accuracy was far lower than expected, suggesting improper fitting. With KPCA, small improvements were observed with the inclusion of the audio data. In the interpolated 10-state dataset, accuracy was improved by 0.8%; the subsampled 10-state dataset showed a 0.4% improvement; and the interpolated 50-state dataset showed a 0.8% improvement. We speculate that audio improves performance because some of the states included the use of loud noises like fans or falling objects hitting the ground to make noise in an attempt to distract the dog. Audio signals also captured bark information from the dog directly. Unfortunately, we saw that the inclusion of environmental data degraded performance to a large extent in both of the 10-state datasets, by about a 10% reduction in the interpolated and 5% reduction in the subsampled datasets. This suggests that the environmental information confused the KPCA model.

The Conv-LSTM model performed better for the subsampled datasets while the KPCA model performed better on the interpolated datasets in every case tested. Because the KPCA model does not require extensive training like the LSTM model, it is generally more useful. The main drawback of KPCA is that it will not work on larger datasets because it requires an expensive eigendecomposition step [35]. During these trials, the subsampled datasets provided too many samples and had to be downsampled to run. As this expanded the general database, KPCA became less useful. A future direction is to consider iterable KPCA [35,36] to remediate this problem. Neural network-based Conv-LSTMs do not have this problem. While training will take proportionally longer with more data, memory usage will remain constant.

**Figure 14:**
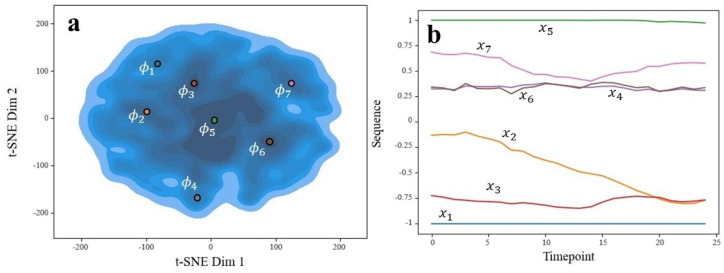
Generated sequences’ latent space (**a**) and corresponding sequences (**b**) indicated by color. Ran on subsample set.

**Figure 15:**
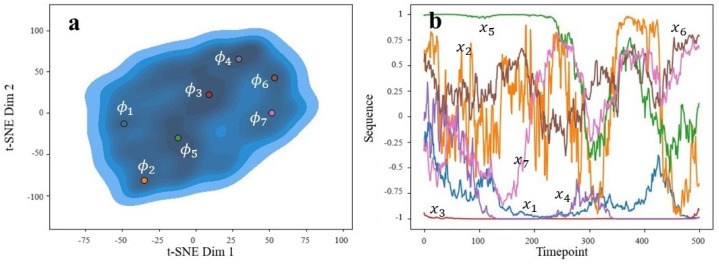
Generated sequences’ latent space (**a**) and corresponding sequences (**b**) indicated by color. Ran on interpolation set.

The autoencoder’s latent space varied as either 4- or 8-dimensional, depending on whether interpolation or subsampling was used, respectively. In both cases, we used a 2-dimensional t-distributed stochastic neighbor embedding procedure [37] to visualize the distribution of latent variables. Ideally, we would have had multiple separate clusters to correspond to distinct actions caught by the sensors. However, this was not observed and instead, all of the data aggregated into one large cluster (see part (a) of Figure 14 and Figure 15). Despite this, we could plot the densities of the embeddings to find regions of relative high density. Recalling the pre-image problem, we could set $\phi^{*}$ to the centers of these high-density regions. The decoder of our autoencoder could then act as the functional inverse from Φ to *X*. After decoding, we obtained the corresponding *x* sequences, which are shown in part (b) of Figure 14 and Figure 15. For simplicity, we only show the *x*-axis acceleration patterns.

We can see that for the subsampled data (Figure 14b), common sequences are fairly simple, consisting of constant lines or up/down linear trends. Constant left or right accelerations are common, such as $x_{1}$, $x_{3}$, $x_{4}$, $x_{5}$, and $x_{6}$. Because of this, direction changes are rarely represented. This makes sense considering these time frames are very short, representing subsecond motion. Interestingly, there is no sequence that goes through the centerline (abscissa = 0), suggesting that the dog either remains on the left or right-half of motion for these sequences. It is uncommon for these short durations to show a change in direction.

For the interpolated data (Figure 15b), the corresponding sequences are noisier and with distinct regions of climb/decline or stability. These are more interesting and expected of canine behaviors. For instance, we do see sequences that pass through the centerline (abscissa = 0), suggesting directional changes. Each sequence found represents a unique motion, which we speculate as follows (for orientation, we take positive values along the *x*-axis to be “right” and negative values to be “left”):

- $x_{1}$: General left-biased movement;
- $x_{2}$: Chaotic and may indicate undesirable behavioral quick movement in response to a distraction, or possibly an appropriate reaction like a jump onto a table;
- $x_{3}$: Constant left-biased movement;
- $x_{4}$: Similar to $x_{1}$ but quicker;
- $x_{5}$: Strong right bias start, followed by motion (dog may start moving or walking);
- $x_{6}$: Right-biased motion (walking);
- $x_{7}$: Walking that starts left and moves more rightward.

**Figure 16:**
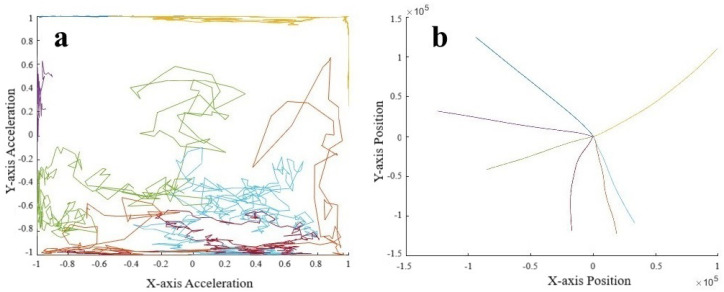
(**a**) Acceleration and (**b**) position sequences derived from $\phi^{*}$ using the decoder from the autoencoder. Color indicates which sequence corresponds to the same sequence in Figure 15.

We look at the acceleration pattern in Figure 16a, where we see that many patterns remain along boundaries of the unit cube, suggesting that these motions correspond to constant directions of travel. By doubly integrating the acceleration data, we can estimate the position in Figure 16b. We notice that movement in the negative x and especially y directions was important enough to warrant separate high-density clusters.

### Potential Outcomes for Guide Dog Behavior Assessments

Due to the controlled environmental conditions, the environmental sensor data were not as helpful for either model type, and potentially confused the models sometimes, resulting in lower accuracies. We speculate that this was due to the IFT being conducted in the same indoor testing location for all dogs, meaning that recorded differences in temperature or light are more likely to be the result of random minor fluctuations rather than significant information. The environment is constant for each state since the entire IFT was conducted in the same room. Prior studies showed that these smart collars are capable of distinguishing environments [16]. However, these tests were performed with significantly different environments, such as indoor vs. outdoor. Therefore, adding the environmental sensors to the data analysis may be more beneficial when the guide dogs undergo tests with highly variable environments.

The Sankey diagram (Figure 11) shows that some states are commonly confused from multiple other states and serve as larger aggregate states, such as “Sees distraction dog” and “Exam complete”. Noticeably, however, many of states are uniquely determined correctly, such as “Idle” and the “Fan passes”. Fortunately, a good number of well-defined states correspond to behavior-related tests, such as the following:

- Passive tests—“Explore-Enters Space” and “Ignore Dog”;
- Noise tests—“Vacuum on-On leash” and “Noise can shake”;
- Dog Distraction tests—“Dog dist-Dog returns to handler”;
- Stranger Fear/Aggression —“Sees Unusual Person”.

The uniqueness in these temperament-related tasks indicates that we are properly able to identify different states that best predict dog temperament of interest. With this in mind, this study is still a preliminary work. We focus on establishing a baseline in this study and will be assessing dog-specific reactions to these events in a future work. Despite this, we observed that the correct identification of certain known IFT states is, in some cases, tantamount to identifying dog-specific temperament. As an example, our models can distinguish between “Explore-Enters Space”, where the dog is active, from “Ignore dog”, where the dog is passive. Then, if a potential guide dog shows similar results in both states, we could infer either attention-seeking (movement when being ignored) or fear (not exploring the space). In this way, a next step for our models would be to distinguish dog temperaments directly from the collar data, rather than relating each through the IFT states.

We also observed that not all states were easily identifiable. Seven of the fifty states mapped to “null”, meaning that they could not be consistently identified from the models, and others mapped to one similar state. For instance, we see several larger aggregate states around “Sees distraction dog” and “Exam complete”. From our findings, as potential improvements for future IFT tests, we recommend removing stand-alone null-mapped states, such as “Rolly toy seen” or “Sees bat”, as these states are difficult to identify from the data. Some of the aggregate states indicate too much similarity between subdivided tasks. The “Exam-load on table”, “Dog on table”, “Vet arrives”, and “Exam complete” states, for instance, could be grouped into one “Exam” superstate, reducing the total number of test states. These recommendations could help simplify the standard IFT testing and data analysis.

Of our main hypotheses (outlined earlier), we have successfully accurately modeled the IFT states to set a baseline for identifying individual dog-specific behavior, and the models could distinguish states that are tantamount to behavior. We leave the analysis of individual effects as a future endeavor, requiring model comparison with the guide dog school’s corresponding BCLs. We were also able to successfully apply multi-sensor data fusion models to better understand dog actions and behavior. This would be an important asset when the IFT testing is performed under different environmental conditions. Unfortunately, the large groupings observed in the AE latent space suggests that common behaviors are not easily distinguishable, and a common lexicon cannot be established. However, higher-density regions suggest that this may be possible in the future with additional data or greater preprocessing measures.

## 6. Conclusions

This study presents our initial results in analyzing the multi-sensor data acquired from a wearable collar system designed for guide dog training. The system contains behavioral and environmental sensors, which were deployed on hundreds of guide dog puppies undergoing training at a prominent guide dog school in the US over the past couple of years. The In-for-Training protocol conducted at Guiding Eyes for the Blind aims to observe potential guide dogs and assess their suitability for future training at the school. In this paper, we developed two supervised methods, a Conv-LSTM network and KPCA, for a preliminary analysis of these data. We found that the Conv-LSTM network worked much better on subsampled data and that KPCA worked better on interpolated data. KPCA is a much faster method since training is not required, but will not work with larger datasets. We found that the IMU accounts for the vast majority of predictability, though audio data improved performance slightly in most datasets. We also created a lexicon of data patterns using an unsupervised autoencoder. We found several regions of relatively higher density in the latent variable space that correspond to more common patterns. This preliminary analysis showed the feasibility of deep learning and Kernel methods in learning specific guide dog states for objective future testing and revealed a starting point for common dog-specific patterns observable through cluster analysis.

## Figures and Tables

**Table 1:** Dataset variants.

Index	Sampling	N States	Fusion Method	Train Size	Test Size
1	interpolate	10	IMU	3159	1355
2	interpolate	10	IMU-Audio	3159	1354
3	interpolate	10	IMU-Audio-Env	1898	814
4	subsample	10	IMU	63,196	27,084
5	subsample	10	IMU-Audio	63,182	27,078
6	subsample	10	IMU-Audio-Env	37,968	16,272
7	interpolate	50	IMU	15,080	6464
8	interpolate	50	IMU-Audio	15,029	6441
9	interpolate	50	IMU-Audio-Env	NaN	NaN
10	subsample	50	IMU	301,616	129,264
11	subsample	50	IMU-Audio	300,580	128,820
12	subsample	50	IMU-Audio-Env	NaN	NaN

**Table 2:** LSTM results. Top results in each section for each metric are in bold.

Dataset	Mac-Prec	Mac-Recall	Mac-F1-Score	Acc
interp 10 IMU	0.363	0.312	0.349	0.297
interp 10 IMU-Audio	0.348	**0.314**	0.337	**0.320**
interp 10 IMU-Audio-Env	**0.459**	0.193	**0.468**	0.283
subsam 10 IMU	0.373	0.375	0.374	0.377
subsam 10 IMU-Audio	0.385	0.389	0.387	0.390
subsam 10 IMU-Audio-Env	**0.442**	**0.413**	**0.419**	**0.468**
interp 50 IMU	**0.057**	**0.051**	**0.090**	**0.052**
interp 50 IMU-Audio	0.017	0.020	0.034	0.017
interp 50 IMU-Audio-Env	NaN	NaN	NaN	NaN
subsam 50 IMU	**0.131**	0.144	**0.139**	0.150
subsam 50 IMU-Audio	0.126	**0.146**	0.136	**0.153**
subsam 50 IMU-Audio-Env	NaN	NaN	NaN	NaN

**Table 3:** KPCA results. Top results in each section for each metric are in bold.

Dataset	Mac-Prec	Mac-Recall	Mac-F1-Score	Acc
interp 10 IMU	0.420	**0.435**	0.416	0.430
interp 10 IMU-Audio	0.429	0.433	**0.420**	**0.438**
interp 10 IMU-Audio-Env	**0.564**	0.237	0.250	0.327
subsam 10 IMU	0.236	0.240	0.208	0.244
subsam 10 IMU-Audio	0.244	**0.246**	**0.216**	**0.248**
subsam 10 IMU-Audio-Env	**0.449**	0.119	0.123	0.195
interp 50 IMU	0.141	0.149	0.138	0.154
interp 50 IMU-Audio	**0.146**	**0.155**	**0.146**	**0.162**
interp 50 IMU-Audio-Env	NaN	NaN	NaN	NaN
subsam 50 IMU	**0.067**	**0.060**	0.051	**0.062**
subsam 50 IMU-Audio	0.056	0.055	**0.057**	0.058
subsam 50 IMU-Audio-Env	NaN	NaN	NaN	NaN

## Data Availability

The data presented in this study are available on request from the corresponding author.
